# Feeling the Stress: Salivary Cortisol Responses of Softball Umpires during National Championships

**DOI:** 10.3390/sports12050128

**Published:** 2024-05-09

**Authors:** Ronald J. Houison, Andrea Lamont-Mills, Michael Kotiw, Peter C. Terry

**Affiliations:** 1 School of Psychology and Wellbeing, University of Southern Queensland, Toowoomba 4350, Australia; peter.terry@unisq.edu.au; 2 Academic Affairs Administration, University of Southern Queensland, Ipswich 4305, Australia; andrea.lamont-mills@unisq.edu.au; 3 School of Health and Medical Sciences, University of Southern Queensland, Ipswich 4305, Australia; mike.kotiw@unisq.edu.au

**Keywords:** stress, anxiety, softball, sports officials, umpires, biochemistry, hormone, cortisol

## Abstract

Stress research in sports tends to focus on athletes, with sports officials typically being overlooked. In the current study, baseline, pre-game, and post-game cortisol levels among a sample of softball umpires were measured to assess the pattern of stress responses and determine if umpire performance (pass/fail) and position on the diamond (plate/field) could be predicted from cortisol levels. Nine male and four female participants aged 25–68 years (*N* = 13, *M* = 47.06 ± 15.65 years) each provided saliva samples on multiple occasions prior to and after officiating games at two Australian National Softball Championships. Data from 65 games were analysed. Performance was assessed using Softball Australia’s official umpire assessment tool. Cortisol levels increased significantly from baseline to pre-game (*p* < 0.001, *d* = −0.69) and declined significantly from pre-game to post-game (*p* < 0.001, *d* = 0.47). Umpiring performances were correctly classified as pass or fail from baseline and pre-game cortisol levels in 61.5% of cases and umpire position on the diamond from pre-game cortisol in 63.1% of cases. Findings suggest that stress management strategies should be recommended to softball umpires for performance enhancement and to safeguard their mental health.

## 1. Introduction

Stress has been defined as “psychological and physical strain or tension generated by physical, emotional, social, economic, or occupational circumstances, events, or experiences that are difficult to manage or endure” [1] (p. 735). Sport participants, who typically operate in a complex environment involving multiple players and fast-developing situations, may see their role as demanding and difficult to manage, thereby causing stress which may influence their performance [2]. Sports officials (i.e., referees, umpires, or judges), like athletes, also operate in the same complex environment, performing under the pressure of expectation from participants, fans, and sponsors in complex situations, which often leads to heightened physical and cognitive load [3], and can be interpreted as stress. Despite this shared performance context, few studies into the effects of stress on sporting officials, and in particular baseball and softball umpires, have been published [4,5]. This paper seeks to address this by exploring the effects of acute stress on the performance of softball umpires.

Stressors, such as psychological factors [6], athletic competition, and physical exertion [7], cause a systemic reaction to occur involving the stress system of the brain and peripheral components, including the activation of two physiological systems that work to prepare the body for action and maintain homeostasis: the hypothalamic–pituitary–adrenal (HPA) axis and the sympathetic–adrenal–medullary (SAM) system [8,9]. Activation of the HPA axis results in the production of cortisol, a glucocorticoid, from the cortex of the adrenal glands [10]. Cortisol plays a key role in the body’s response to physiological and psychological stressors by modulating metabolism and increasing energy available to the body and the brain [10,11]. The pituitary gland, stimulated by corticotropin-releasing hormone (CRH) from the hypothalamus, produces adrenocorticotropic hormone to regulate cortisol levels. CRH is released cyclically by the hypothalamus, resulting in cortisol levels that are elevated in the morning and low in the evening [12,13]. Catecholamines, such as adrenaline, are produced following the activation of the SAM system and stimulate the body’s metabolic and cardiovascular systems. These catecholamines have a short half-life of only minutes and are not stable at room temperature, making sample storage impractical [8] and hence less attractive than other stress markers for use in field-based research.

Cortisol is considered a primary physiological marker of HPA axis activity and an indicator of stress [14]. Research into stress has been conducted using self-report questionnaires, steroid (cortisol) concentration using saliva [15], and physical exertion via heart rate monitors and GPS systems [16]. Tests that minimise additional stress, such as being minimally invasive and quick to administer, and which provide accurate and unbiased results, are required. As such, biological stress assessment using cortisol from saliva samples is typically preferred [17]. The concentration of cortisol in saliva has been shown to positively correlate with the level of stress experienced by the individual [7] and is a seen as a reliable and accurate measure of an individual’s stress level [17]. Up to 95% of secreted cortisol will be bound to proteins in the bloodstream and will not be available for analysis. Only unbound, or free, cortisol is thought to be biologically active and enters cells easily due to its low molecular weight and lipophilic nature, meaning that the concentration of free cortisol can be measured in bodily fluids, such as sweat, urine, tears, and saliva [18]. While all of these fluids can be collected, saliva is the most easily collected, and this can be done without adding stress [17]. The concentration of cortisol in saliva has also been found to accurately reflect the concentration of unbound cortisol in blood and plasma samples [18]. As saliva sampling is not invasive and takes less than two minutes [18], it is well-suited for use in the sporting context where time to collect data is often seen as an imposition.

Salivary cortisol is stable at room temperature, increasing the length of time samples remain viable [8], which is an important consideration in field-based research where access to refrigerators or freezers may be restricted. Cortisol concentration in samples awaiting analysis also remains stable during refrigerated storage. For example, Garde and Hansen [19] reported that saliva could be stored for up to three months at 5 degrees Celsius, and up to twelve months at −20 to −80 degrees Celsius without detectable decreased cortisol concentration. Samples could be thawed and refrozen up to four times while maintaining viability. The analysis of cortisol in saliva samples can be achieved using various assay techniques, which include radioimmunoassay, enzyme-linked immunosorbent assay (ELISA), automated electrochemiluminescence assays, and liquid chromatographic methods coupled with mass spectrometry (LC-MSMS). Most assays are conducted using immunoassay methods due to their convenience and the relative scarcity of LC-MSMS technology-equipped laboratories [20]. In healthy adults, cortisol levels have been reported to range between 10.2–27.3 ng/mL in the morning and 2.2–4.1 ng/mL at night [19].

### 1.1. Stress and Health

The activation of the stress management systems helps the body to perform in stressful situations and afterwards regain homeostasis [9]. Turner and associates [21] conducted a systematic review of research on the health impact of high (exaggerated) and low (blunted) stress reactivity. After reviewing 47 papers that included more than 32,000 participants, they reported that an intermediate-level stress response tended not to result in adverse health outcomes, whereas exaggerated and blunted reactivity predicted health and disease outcomes in 32% of all reported findings. Exaggerated stress reactivity predicted increased risk of cardiovascular disease, while blunted stress reactivity predicted increased illness frequency, obesity, depression, and anxiety.

### 1.2. Stress and Performance

Research into the cortisol–performance relationship has been conducted with athletes (e.g., tennis [7], badminton [22], taekwondo [23], and soccer [24]), musicians [25], and academics [8]. The relationships reported between cortisol and performance have varied. In tennis, higher concentrations of cortisol were found in losers than in winners both prior to and post-performance [7]. Greater increases in cortisol levels were reported in losers than winners after the completion of competition in tennis and badminton [7,22]. In taekwondo, no difference in cortisol was recorded between winners and losers in samples taken 30 min before and after competition [23]. In female soccer players, an increase in cortisol was detected prior to a match (anticipatory rise), but no variation in cortisol level was recorded based on the outcome of the match [24]. Among musicians, cortisol levels before and after a performance were reported as higher than levels reported on a neutral day, with the rise in cortisol prior to a performance attributed to performance anxiety [25]. In stress associated with academic activities such as oral presentations, cortisol levels were reported to be higher after a presentation rather than before. [8]. Variation in the results in sport could be associated with the level of complexity of different types of performances, contact versus no/low contact, and the physical demands placed upon the participants [7].

Some utility in predicting athletic performance from cortisol levels has been reported. In taekwondo competitors, a positive correlation was identified between cortisol level during competition and performance. A stepwise multiple regression analysis reported that 25.5% of the performance variance was predicted by the cortisol level recorded during competition [23]. In a controlled environment exploring the influence of cortisol on tennis serve performance, an inverse correlation was reported between cortisol and performance, with cortisol explaining 19% of the variance in serve performance [26].

Although much research into the stress–performance relationship among athletes has been published, much less research has been conducted regarding sports officials [27]. Sports officials, while often overlooked, are essential participants in sport [3,27]. They tend to come from the playing or coaching ranks of their sport, often choosing officiating to prolong their sport involvement, to participate at higher levels than possible as a player, or to continue to enjoy the social aspects of sports participation [27]. Officials are required to learn rules and apply them in situations that are often complex and fast-moving, and need to adapt to changing contexts, playing strategies, and physical demands [3]. The expectations of athletes, coaches, and the officials themselves is that officials will correctly adjudicate on situations that arise in the game. This expectation has the potential to create high levels of acute stress which arise from incidents that are short-term and time-limited [28], such as making an officiating error or when physical resources are being overtaxed [29]. This suggests that the activity levels and physical demands on the official may have a bearing on stress levels, in addition to stress resulting from cognitive load. Sport performance can be negatively impacted by acute stress, whilst satisfaction and success in sport relies on the ability to effectively cope with these episodes of acute stress [28]. A key contributor to success is the use of stress-mitigating strategies [29], which suggests that the officials’ stress levels and performances are likely to be related. The combination of increased performance expectations, scrutiny, and criticism highlights a need for more research into the effects of stress on the performance and health of sports officials. Given potential sport differences, Australian softball umpires were selected as the focus of this study.

### 1.3. Softball and Softball Umpires

**Figure 1:**
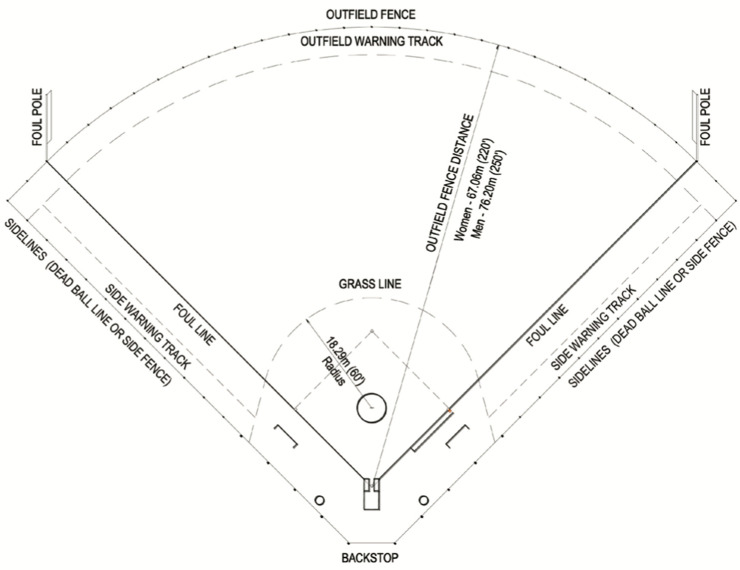
The softball field of play [30]. Used with permission.

Softball is a game played between two teams and is similar to baseball. There are two main versions of softball, fastpitch and slowpitch. This study only considered the fastpitch variant, in which each team has nine defensive players, called fielders, and nine offensive players, called batters, actively engaged in the game at any given time. Both teams may have reserve players who can be used at any time. The playing field has an “infield” in which the key feature is a diamond shape made up of home plate (the point nearest the backstop) and three bases. Beyond the infield to the fence surrounding the playing field is the “outfield,” and between the foul lines and the side fences is an area termed “foul territory” (see Figure 1) [30].

Softball umpires are positioned in various locations on the field and have varying roles. The “plate umpire” is positioned behind home plate and is involved in the majority of the decisions in play, from judging whether or not unhit pitches pass through the strike zone (strikes and balls) to judging whether batters and runners are safe or out on plays, to managing and maintaining the teams’ rosters and substitutions. Base umpires are initially stationed behind bases (located at the remaining three corners of the diamond), and outfield umpires are stationed in the outfield. Base and outfield umpires (referred to in this study collectively as “field” umpires) adjudicate on plays in their general vicinity [31,32].

The role of the plate umpire tends to be more stressful than that of the field umpire. Although little research has been published that explores this, Rainey [4,33] reported that fear of failure and interpersonal conflict were prime sources of stress in baseball and softball umpires. The plate umpire has responsibility for making decisions with every pitch thrown. Incorrectly judging a pitch as a ball or a strike may be perceived as unfair and lead to people (players, coaches, spectators) becoming verbally aggressive and thus increasing interpersonal conflict [34]. These perceived errors in judgement, particularly in important games, may increase the umpire’s fear of failure [4]. Field umpires adjudicate on plays less frequently than plate umpires, and while these decisions may also precipitate interpersonal conflict or fear of failure, it is expected that lower stress levels would result because of the lower frequency of decisions made.

Softball umpires are relatively sedentary compared to officials, such as Australian Football League (AFL) umpires, who may cover more than 10 km during a game [35]. Hence, the stress levels of softball umpires should be compared primarily with research findings of other sedentary activities such as academic presentations [8] or musical performance [25], and to a lesser extent with findings related to non-contact sports [7,15]. As the impact of physiological activity on stress levels is well-recognised to have a significant influence on cortisol levels, comparison with similar activities is required. Based on those prior findings, it was hypothesised that participants in the present study would show increased cortisol levels from baseline to pre-game (H1), that cortisol levels would subside post-game (H2), that participants whose umpiring performances were rated as a fail (i.e., made more errors) would show higher pre-game cortisol levels than for those performances rated as a pass (i.e., less errors) (H3), and that cortisol levels would be higher for plate umpires than field umpires (H4).

## 2. Materials and Methods

### 2.1. Participants

Participants officiating at two Softball Australia under age 2020 national championships (*N* = 13, *M* = 47.06 ± 15.65 years) were recruited from among the 21 umpires appointed. Nine male (*M* = 53.20 ± 14.69 years) and four female (*M* = 35.25 ± 9.29 years) participants aged 25–68 years old were recruited, representing 61.9% of the population of interest. Five participants officiated at one of the championships and eight participants officiated at the other. The umpire accreditation levels of the participants ranged from Level 4 to Level 8 (*M* = 5.73 ± 0.67). Australian umpire accreditation ranges from Level 1 to 8, with the higher-numbered level being more highly qualified. Levels 7–8 are awarded for contributions to the umpiring program [36], which usually involves achieving international accreditation from the World Baseball Softball Confederation (WBSC) [37] and having completed appointments to officiate at WBSC world championships. Levels 5–6 are assessed via theory exam and practical exams at national competitions. Levels 2–4 are assessed via theory exam and practical exams at state competitions, and Level 1 requires a theory exam pass with no formal assessment [36]. Years of umpiring experience ranged from 7 to 30 years (*M* = 18.78 ± 6.77 years). Participation in the study was voluntary with no incentives provided.

### 2.2. Measures

Cotton wool balls and disposable paper cups were used to collect baseline, pre-game, and post-game saliva samples, and the samples were stored in sterile Corning 2.0 mL micro centrifuge tubes with screw cap (Corning product number 403915). Samples were stored in a portable cooler containing frozen freezer bricks until they were transferred to a domestic freezer (−20 °C) at the end of each day’s competition.

The performances of all championship umpires, including the participants, were assessed as part of normal championship procedures. The assessment was conducted by the umpire management teams, consisting of the Umpire-in-Chief and two assessors. All umpire management team members had achieved international-level umpiring qualifications and experience and were accredited by Softball Australia as senior assessors. The management teams were appointed to the championship by Softball Australia. Quantification of performance was undertaken using performance data that included participant name, umpiring role, assessment categories to which performance observations related, the observed behaviours of the participant, and a flag indicating whether the recorded entry was positive or negative.

Umpire performance in each game was evaluated by a member of the management team using Softball Australia’s umpire assessment tool [38]. Individual umpire assessments per game are part of the normal process that occurs at each championship. Five assessment areas are assessed: General, Game Control, Judgement and Rules, Positioning and Calls, and Plate Work. Each area has several specific categories reported: General = 6, Game Control = 7, Judgement and Rules = 2, Positioning and Calls = 3, Base Mechanics = 2, and Plate Work = 3. Marks are assigned in each category on a scale of 1–5, in which the candidate scored 1 if they failed to comply with more than 4 requirements of this category and scored 5 if the umpire did everything that the category required and also displayed exceptional umpiring ability and skill. The default score is 4, where the umpire is deemed to have provided a satisfactory performance in each category [38].

Umpire performance is based on a “penalty”-style assessment in which participants lose marks on an “errors made” basis. Participants are penalised for the first error observed and every second error thereafter (i.e., on the first, third, and fifth errors). A category score of 5 is awarded only when no errors were attributed to the category and the umpire is subjectively judged to have performed at an exceptional standard. An umpire’s performance score for each game is determined by calculating the raw score totals in each assessment area, which is then converted to scale marks using a conversion chart. Scaled area scores are totaled with the total scaled score used to assess pass or fail. Higher total scores signify better performance. Game pass marks are assigned to each accreditation level and position/role on-diamond (i.e., plate umpire or base/field umpire): Level 4: Plate = 80, base = 61; Level 5–8: Plate = 82, base = 61 [38].

### 2.3. Procedure

The study was conducted over two, one-week periods in 2019 and 2020. Prior to the championships, all appointed umpires were invited to participate in the study by Softball Australia via email. Recruited participants provided written consent and demographic information (sex, age, accreditation level, and years of experience) at umpire meetings prior to the commencement of the championships.

Baseline salivary cortisol samples were collected at scheduled pre-competition umpire meetings the day before each championship commenced. Meeting times and participant availability were subject to arrival times of interstate-based participants and the scheduling of the meetings by the tournament Umpire-in-Chief. Baseline samples were provided by participants officiating at one championship at approximately 7:50 p.m., and by participants officiating at the other championship at approximately 3:50 p.m. Saliva samples were collected from each participant before and after every game in which they officiated. Pre-game and post-game samples were collected 30 min prior to the scheduled start of a game and 30 min after the completion of a game. The participants were asked to abstain from eating, smoking, or drinking (except for water) and not to brush their teeth during the 60 min before the pre-game saliva samples were collected, and in the 30 min before post-game samples were collected.

Each participant officiated in between 7 and 13 games, and 271 samples in total were collected, comprising 13 baseline samples, 131 pre-game samples, and 127 post-game samples. Participants provided the game-day samples in an alcove adjacent to the Umpire-in-Chief’s office on the lower level of the softball stadium, a relatively quiet and private environment. If umpires were appointed to consecutive games, one sample was collected between the two games as both a post-game and pre-game sample. For each sample collected, participants were provided with an unused paper cup and cotton wool ball. The participant placed the cotton ball into their mouth, gently chewed to stimulate salivation until the cotton was saturated with saliva, and then deposited the cotton ball in the paper cup. The saliva sample was squeezed from the cotton ball into the paper cup and transferred to the micro centrifuge tube for storage by the first author. Samples of 50 microliters or more were deemed to be sufficient [39]. The tube was tagged with a unique number which was logged in FreezerPro [40]. Samples were kept on ice until transferred to a domestic freezer at the end of each day and stored at −20 degrees Celsius.

Following the championships, the samples were transferred on ice in a cooler box to the −80-degree-Celsius freezer on campus at the University of Southern Queensland (UniSQ), Toowoomba campus for storage pending assay. Samples were analysed by qualified staff using commercial enzyme-linked immunosorbent assay (ELISA) kits [Cortisol Competitive Human ELISA kit (EIAHCOR)] with an analytical sensitivity = 17.3 pg/mL, assay range = 100–3200 pg/mL, and a sample volume of 12.5 µL [39]. After thawing, samples were assayed in accordance with the process recommended by the test manufacturer [41]. Every sample was assayed in duplicate to ensure reliable cortisol data.

Members of the umpire management team documented each umpire’s performance in every game on Excel spreadsheets in accordance with Softball Australia’s procedures [42]. After the championships, the first author used the umpire assessment tool [38] to calculate the participants’ performance from data in the spreadsheets, which were provided to the research team by Softball Australia’s Umpire-in-Chief (Development).

### 2.4. Ethics

The UniSQ Human Ethics Committee approved the study on 19 November 2019. Material Assessment Approval #21BIO012A for the assay of human biological material (saliva) was granted by the UniSQ Facilities Management on 15 October 2021. The recruitment of participants was conducted, and written consent obtained at umpire meetings at both championships. As part of the informed consent process, participants were allocated a randomly generated number (random.org) which was used as a unique identifier (ID) to ensure the confidentiality of participant data. All participants were 18+ years of age when consent was provided.

### 2.5. Statistical Analysis

SPSS for Mac, Version 29, IBM Corporation, Armonk, NY, USA [43] was used to conduct all statistical analyses. Firstly, the correlation between the pre-game cortisol level and the time of day that the sample was obtained was calculated to determine if the diurnal variation in cortisol level would be a confounding variable. Next, descriptive statistics were calculated for all participant cortisol concentrations at baseline, pre-game, and post-game. Descriptive statistics were calculated for years of experience and the correlation between the years of experience and game result was calculated to determine if experience would affect game result. Paired samples *t*-tests were performed to test for statistically significant differences between the overall means of baseline and pre-game cortisol levels, baseline and post-game cortisol levels, and pre-game and post-game cortisol levels. Next, independent samples *t*-tests were used to compare pre-game cortisol levels by umpire rating (pass/fail) and position on the diamond (plate/field). Discriminant function analysis (DFA) was used to quantify the percentage of correct classifications of umpire rating (pass/fail) and position on the diamond (plate/field) from baseline, pre-game, and post-game cortisol levels. One of the assumptions underlying DFA procedures is that all measures are independent. Although we combined multiple cortisol samples from the same participants into a single dataset, thereby treating them as independent data points, DFA has been shown to be robust even when underlying assumptions are not met [44]. Effect sizes in the form of Cohen’s *d* were interpreted as small (0.20), moderate (0.50), or large (≥0.80) [44].

### 2.6. Data Screening

Cases from 13 participants were screened for invalid values (≤0 ng/mL) and missing values. Forty-three cases were discarded due to invalid values, and thirteen cases were discarded due to missing values. One participant’s results were deemed to be an outlier and discarded due to an abnormally large range of measures, from 0.84 ng/mL through to 59.15 ng/mL, with a mean of 16.48 ng/mL. Following data screening, there were 66 cases from eight participants that went forward for analysis. Upon review, significant deviation from univariate normality was evident for pre-game mean cortisol levels. One further case was identified as an outlier and removed. The remaining 65 cases showed univariate normality with skewness = 0.31 (baseline), 0.88 (pre-game), and 1.18 (post-game), and kurtosis = −0.56 (baseline), 0.64 (pre-game), and 1.25 (post-game).

## 3. Results

### 3.1. Cortisol Levels and Time of Day

Given that time of day has been implicated as a potential confounding variable in the assessment of cortisol [45], a Pearson correlation coefficient was calculated to quantify the relationship between pre-game cortisol level and the time that the sample was provided. A very small correlation was found between the two variables, *r*(63) = 0.09, *p* = 0.49, meaning that the time that the sample was provided explained less than one percent (0.81%) of the variance in cortisol levels.

### 3.2. Group Cortisol Levels

The collective mean cortisol levels for all umpires across all games were assessed, comparing baseline level with pre-game level, baseline level with post-game level, and pre-game level with post-game level (Table 1). As a group, participants recorded an increase in mean cortisol level of 40.31% between the baseline and pre-game samples, followed by a decrease between mean pre-game and post-game cortisol level of 29.46%. The mean post-game cortisol level was 15.38% above the mean baseline level.

**Figure 2:**
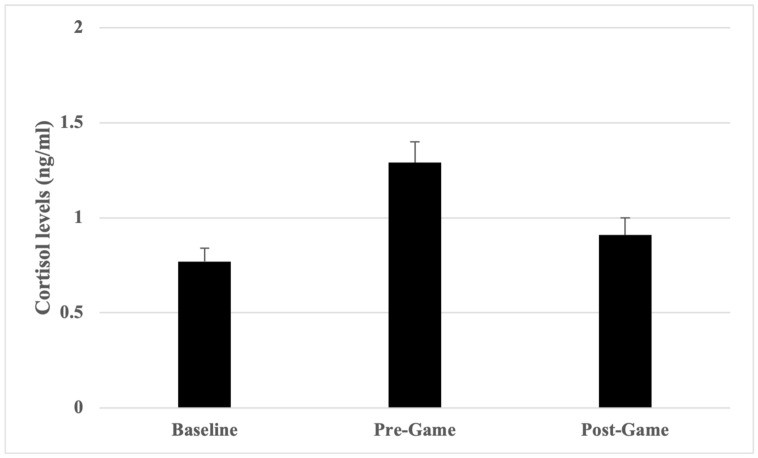
Group cortisol levels (mean ± SE).

Repeated measures *t*-tests showed that cortisol levels increased significantly from baseline to pre-game, *t*(64) = −5.23, *p* < 0.001, *d* = −0.69 (moderate effect), and then subsided significantly from pre-game to post-game, *t*(64) = 3.81, *p* < 0.001, *d* = 0.47 (moderate effect), although it remained above baseline. There was no significant difference in cortisol level between baseline and post-game, *t*(64) = −1.63, *p* = 0.11, *d* = −0.23 (small effect) (Figure 2).

### 3.3. Umpire Performance

Mean years of experience was calculated for participants in pass and fail game result groups (Table 2). A Pearson product correlation was calculated to quantify the relationship between the variables and a very small, non-significant positive correlation was found, *r*(63) = 0.05, *p* = 0.71, meaning that the years of experience explained less than one percent (0.25%) of the variance in umpire performance.

It is apparent that cortisol levels were higher for umpiring performances assessed as fail than performances assessed as pass, at all three timepoints (Table 2). Independent samples *t*-tests indicated that the observed differences were not statistically significant, at baseline [*t*(63) = 1.05, *p* = 0.15, *d* = 0.29 (small effect)], pre-game [*t*(63) = 1.16, *p* = 0.13, *d* = 0.32 (small effect)], and post-game [*t*(63) = 1.25, *p* = 0.11, *d* = 0.35 (small effect)]. When umpiring performances were dichotomised into pass (*n* = 47) and fail (*n* = 18) categories, DFA showed that performance could be correctly classified as pass/fail from baseline, pre-game, and post-game cortisol levels with 60.0% accuracy (Wilks’ *λ* = 0.967, *p* = 0.56). Correct classifications increased to 61.5% when only baseline and pre-game cortisol levels were included in the DFA, despite not reaching significance (Wilks’ *λ* = 0.974, *p* = 0.45).

For umpiring performances that were assessed as a pass, significant differences were found when comparing baseline to pre-game levels, *t*(46) = −4.43, *p* < 0.001, *d* = −0.71 (moderate-to-large effect) and comparing pre-game to post-game levels, *t*(46) = 2.99, *p* = 0.004, *d* = 0.50 (moderate effect). No significant difference was found when comparing baseline to post-game levels, *t*(46) = −1.15, *p* = 0.26, *d* = −0.20 (small effect, Table 2).

**Figure 3:**
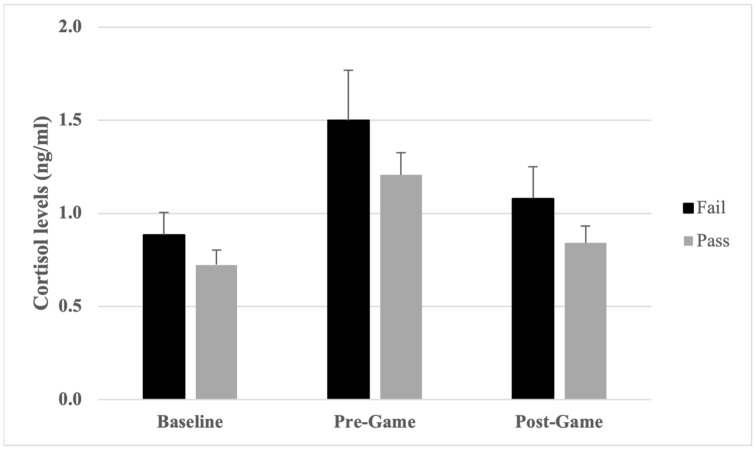
Cortisol levels (mean ± SE) for umpire performances assessed as fail and pass.

For umpiring performances that were assessed as a fail, significant differences were found when comparing baseline to pre-game cortisol levels, *t*(17) = −2.76, *p* = 0.013, *d* = −0.70 (moderate-to-large effect) and pre-game to post-game cortisol levels, *t*(17) = 2.41, *p* = 0.028, *d* = 0.43 (small-to-moderate effect). No significant difference was found when comparing baseline to post-game cortisol levels, *t*(17) = −1.23, *p* = 0.24, *d* = −0.31 (small effect, Table 2). Results for umpire performance are shown graphically in Figure 3.

### 3.4. Umpire Position on the Diamond

Cortisol levels were higher for plate umpires than field umpires at pre-game and post-game timepoints, although the reverse was true at baseline (Table 3). Independent samples *t*-tests indicated that the observed differences were not statistically significant, at baseline [*t*(63) = −1.03, *p* = 0.15, *d* = 0.27 (small effect)], pre-game [*t*(63) = 1.08, *p* = 0.14, *d* = 0.29 (small effect)], and post-game [*t*(63) = 0.96, *p* = 0.17, *d* = 0.25 (small effect)]. When position on the diamond was dichotomised into plate (*n* = 22) and field (*n* = 43) categories, DFA results showed that position could be correctly classified from baseline, pre-game, and post-game cortisol levels with 60.0% accuracy (Wilks’ *λ* = 0.923, *p* = 0.18). Correct classifications increased to 63.1% when only pre-game cortisol levels were included in the DFA, despite not reaching significance (Wilks’ *λ* = 0.982, *p* = 0.28).

**Figure 4:**
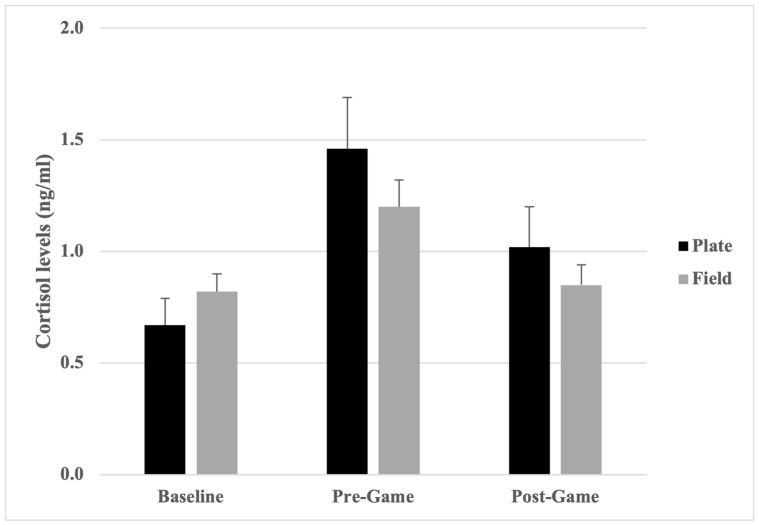
Cortisol levels (mean ± SE) for plate and field umpires.

When comparing stress levels over time, significant differences were found for plate umpires for all three timepoint comparisons: baseline to pre-game: *t*(21) = −4.59, *p* < 0.001, *d* = −0.91 (large effect), baseline to post-game: *t*(21) = −2.25, *p* = 0.04, *d* = −0.49 (small-to-moderate effect), and pre-game to post-game: *t*(21) = 2.17, *p* = 0.04, *d* = 0.72 (moderate effect). Field umpires showed significant differences from baseline to pre-game, *t*(42) = −3.24, *p* = 0.002, *d* = −0.56 (moderate effect) and pre-game to post-game, *t*(42) = 3.14, *p* = 0.003, *d* = 0.58 (moderate effect). The baseline to post-game comparison was not significant for field umpires, *t*(42) = −0.33, *p* = 0.75, *d* = −0.05 (small effect). Results for plate and field umpires are shown graphically in Figure 4.

## 4. Discussion

In the present study, saliva samples were collected from softball umpires officiating at two Australian national championships for the purpose of assessing their stress levels. The concentration of cortisol in the samples was collected and analysed consistently with previous studies of athletes [7,15], musicians [25], and academics [8]. The present study also assessed whether cortisol levels were predictive of umpiring performance and assessed the effects of umpire position on the diamond on cortisol levels. As hypothesised, participants recorded an increase in cortisol levels from baseline to just before officiating a game (H1), cortisol levels subsided post-performance to a level above the baseline (H2), and cortisol levels were predictive of umpiring performance (H3) and umpire position on the diamond (H4).

The diurnal variation in cortisol had the potential to confound results. Cortisol levels tend to peak in the morning and decrease over the course of the day [12,13]. Pre-game samples were obtained from participants between 8:30 a.m. and 6:45 p.m. Given evidence that cortisol levels fluctuate diurnally, it was important to check that time of day was not a confounding variable. The non-significant correlation between time of day and cortisol levels and the fact that less than 1% of the variance was explained support the reliability of the findings.

The significant overall changes in cortisol concentration from baseline to pre-game to post-game supported our hypotheses (H1 and H2). As a group, participants in the study recorded a 40.31% increase in mean cortisol level between the baseline and pre-game samples, which was statistically significant. Also as hypothesised, the participants experienced a statistically significant 29.46% decrease between mean pre-game and post-game cortisol levels. The mean post-game cortisol level was 15.38% above the mean baseline level. The significant increase in cortisol from baseline to pre-game suggests that umpires experience increased stress in anticipation of games, and a decrease in stress post-game in much the same way as found for musicians [25]. Cortisol levels in musicians rose significantly from baseline to five minutes prior to performance and then decreased after the performance [25]. Whether increased stress has a facilitative or debilitative effect on performance [46], be that umpiring or music performance, appears to depend on whether the individual in question labels the somatic signals of the stress response (e.g., increased heart rate, butterflies in the stomach, sweaty palms, etc.) as anxiety that may impede performance or excitement that signals readiness to perform [47].

Regarding umpiring performance, performances assessed as a pass were associated with lower cortisol levels at pre-game and post-game than those that were assessed as fail, with small effects that were not significant. Although not statistically significant, our results showed that umpire performances could be correctly classified into pass or fail categories from baseline and pre-game cortisol values with 61.5% accuracy, supporting our hypothesis (H3). Previous research in tennis found that those who won the match showed a 20% decrease in cortisol level from pre-match to post-match, whereas losing players showed an increase in cortisol from pre-match to post-match [7]. Multiple umpiring performances were assessed for each participant in the present study, and participants who had performances assessed as fail also had performances assessed as pass. Lai et al. [48] proposed that self-efficacy, hope, optimism, and resilience positively influence performance, and it could be argued that successful performances were predicated on these factors. Although softball umpires are less active than many other sports officials, they are required to be standing and mobile for the entirety of every game, and are expected to maintain a level of fitness enabling them to keep pace with the athletes and the game [31]. At the international level, officials are required to achieve a prescribed level of fitness as a condition of appointment to officiate at competitions [49]. Over the course of a championship lasting up to seven days, it can be expected that umpires will experience fatigue and muscle soreness. These stressors, if not well managed, can be factors impacting an umpire’s cortisol levels. Future research should consider assessment of cognitive, physical, and affective states to explore participants’ sources of stress.

When umpire position on the diamond was dichotomised into plate and field categories, plate umpires reported higher pre-game cortisol concentrations than field umpires, with a small effect that was not significant. Pre-game cortisol values could correctly classify umpire position on the diamond with 63.1% accuracy, supporting our hypothesis (H4). Existing research suggests that failure and interpersonal conflict [4,33] are prime stressors for umpires. The plate umpire’s greater involvement in the game than the field umpire provides more opportunity for contentious calls, leading to increased opportunity for conflict with athletes, coaches, and spectators. Softball is often played in summer, and the heat and humidity experienced during games can be stressful, particularly when considering the activity levels and the equipment worn by the plate umpire. The plate umpire is more active than the field umpires and can be observed moving to a semi-squat position to be in the best position to judge the location of the ball in relation to the strike zone for every pitch thrown. Plate umpires wear additional protective equipment, including a facemask (with or without attached helmet) and chest and leg protection. This equipment traps body heat, potentially adding to the stressors experienced by the plate umpire. The added activity and equipment that is unique to the plate umpire and the greater workload help to explain the higher levels of cortisol reported by the plate umpire.

We acknowledge that there are limitations with this study. Although the present sample size was small, previous studies of cortisol levels in performance contexts have also yielded significant results from small samples [7,23,25]. While statistical significance was not achieved in classifying game result or position on the diamond, all effects were in the predicted direction. Broad generalisation of the findings is hampered by the small number of participants, which is a consequence of there being only a small number of umpires officiating at the national level in Australia, and of the fact that COVID-19 restrictions the year following the collection of these data prevented the opportunity for further data collection. However, the significant findings in the present study provide a baseline for future cortisol/stress research with sports officials. The present study focused on only one factor contributing to the performance of softball umpires. Future research could combine assessment of biochemical markers such as cortisol with affective and/or cognitive indicators to increase our knowledge of sports official performance.

The present findings may help to inform future studies regarding the zone of optimal stress associated with optimum performance and long-term health benefits [21]. The results may also be used to inform efforts to improve umpire performance by developing and including stress management interventions in programs run for Softball Australia and the state-based affiliates by sport psychologists. Interventions could be included in existing programs, such as training clinics, pre-assessment events [50], and at national championships. Softball is a popular sport in the USA, with more than 8 million participants in 2021 [51], and softball as a sport has much in common with baseball. Therefore, it is anticipated that the results of this study and the interventions proposed are likely to have widespread relevance in countries with large softball and/or baseball communities.

## 5. Conclusions

Several conclusions can be drawn from the results of the present study. Firstly, softball umpires showed a predictable and significant increase in cortisol levels from baseline to pre-game, and a significant decrease in cortisol levels from pre-game to post-game. Secondly, cortisol levels were found to be somewhat predictive of umpiring performance. Thirdly, cortisol levels were associated with umpire position on the diamond, as plate or field umpire.

## Figures and Tables

**Table 1:** Mean group cortisol levels (ng/mL) at baseline, pre-game, and post-game.

	*N*	Baseline	Pre-Game	Post-Game
Mean (SD)	65	0.77 (0.54)	1.29 (0.91)	0.91 (0.68)

**Table 2:** Cortisol levels (ng/mL) and experience (years) for fail and pass umpire performances.

	*n*	Experience	Baseline	Pre-Game	Post-Game
Fail mean (SD)	18	15.56 (8.36)	0.88 (0.51)	1.50 (1.15)	1.08 (0.74)
Pass mean (SD)	47	16.36 (7.62)	0.72 (0.55)	1.21 (0.81)	0.84 (0.65)

**Table 3:** Cortisol levels (ng/mL) for plate and field umpire positions.

	*n*	Baseline	Pre-Game	Post-Game
Plate mean (SD)	22	0.67 (0.57)	1.46 (1.09)	1.02 (0.83)
Field mean (SD)	43	0.82 (0.52)	1.20 (0.81)	0.85 (0.58)

## Data Availability

Data are available from the first author on request. The data are not publicly available due to the small population and limited number of events from which participants may be recruited, potentially compromising the confidentiality of the participants’ identities.

## References

[1] Colman A.M. (2009). Dictionary of Psychology.

[2] Arnold R., Fletcher D., Arnold R., Fletcher D. (2021). Stressors, hassels, and adversity. Stress, Well-Being, and Performance in Sport.

[3] O’Brien K.A., Rynne S.B. (2020). Seen but not heard: Missing the mark in conceptualizing high performance officiating. Sport Soc..

[4] Rainey D. (1995). Sources of stress among baseball and softball umpires. J. Appl. Sport Psychol..

[5] Rainey D.W. (1994). Stress experienced by baseball and softball umpires. Percept. Mot. Ski..

[6] Dickerson S.S., Kemeny M.E. (2004). Acute stressors and cortisol responses: A theoretical integration and synthesis of laboratory research. Psychol. Bull..

[7] Lautenbach F., Laborde S., Klämpfl M., Achtzehn S. (2015). A link between cortisol and performance: An exploratory case study of a tennis match. Int. J. Psychophysiol..

[8] Tammayan M., Jantaratnotai N., Pachimsawat P. (2021). Differential responses of salivary cortisol, amylase, and chromogranin A to academic stress. PLoS ONE.

[9] Fatouros I., Chatzinikolaou A., Paltoglou G., Petridou A., Avloniti A., Jamurtas A., Goussetis E., Mitrakou A., Mougios V., Lazaropoulou C. (2010). Acute resistance exercise results in catecholaminergic rather than hypothalamic-pituitary-adrenal axis stimulation during exercise in young men. Stress.

[10] Levine A., Zagoory-Sharon O., Feldman R., Lewis J.G., Weller A. (2007). Measuring cortisol in human psychobiological studies. Physiol. Behav..

[11] Hellhammer D.H., Wust S., Kudielka B.M. (2009). Salivary cortisol as a biomarker in stress research. Psychoneuroendocrinology.

[12] Mayo Clinic Test ID: SALCT. https://www.mayocliniclabs.com/test-catalog/overview/84225#Clinical-and-Interpretive.

[13] El-Farhan N., Rees D.A., Evans C. (2017). Measuring cortisol in serum, urine and saliva—Are our assays good enough?. Ann. Clin. Biochem..

[14] Chojnowska S., Ptaszynska-Sarosiek I., Kepka A., Knas M., Waszkiewicz N. (2021). Salivary biomarkers of stress, anxiety and depression. J. Clin. Med..

[15] Filaire E., Alix D., Ferrand C., Verger M. (2009). Psychophysiological stress in tennis players during the first single match of a tournament. Psychoneuroendocrinology.

[16] Blair M.R., Elsworthy N., Rehrer N.J., Button C., Gill N.D. (2018). Physical and physiological demands of elite rugby union officials. Int. J. Sports Physiol. Perform..

[17] Kirschbaum C., Hellhammer D.H. (1989). Salivary cortisol in psychobiological research: An overview. Neuropsychobiology.

[18] Kirschbaum C., Hellhammer D.H. (2000). Salivary cortisol. Encyclopedia of Stress.

[19] Garde A.H., Hansen A.M. (2005). Long-term stability of salivary cortisol. Scand. J. Clin. Lab. Investig..

[20] Inder W.J., Dimeski G., Russell A. (2012). Measurement of salivary cortisol in 2012—Laboratory techniques and clinical indications. Clin. Endocrinol..

[21] Turner A.I., Smyth N., Hall S.J., Torres S.J., Hussein M., Jayasinghe S.U., Ball K., Clow A.J. (2020). Psychological stress reactivity and future health and disease outcomes: A systematic review of prospective evidence. Psychoneuroendocrinology.

[22] Jimenez M., Aguilar R., Alvero-Cruz J.R. (2012). Effects of victory and defeat on testosterone and cortisol response to competition: Evidence for same response patterns in men and women. Psychoneuroendocrinology.

[23] Lautenbach F., Lobinger B.H. (2018). Cortisol predicts performance during competition: Preliminary results of a field study with elite adolescent taekwondo athletes. Appl. Psychophysiol. Biofeedback.

[24] Oliveira T., Gouveia M.J., Oliveira R.F. (2009). Testosterone responsiveness to winning and losing experiences in female soccer players. Psychoneuroendocrinology.

[25] Turan B., Hurst-Wajszczuk K., Edwards D.A. (2022). Hormone and enzyme reactivity before, during, and after a music performance: Cortisol, testosterone, and alpha-amylase. Compr. Psychoneuroendocrinol..

[26] Lautenbach F., Laborde S., Achtzehn S., Raab M. (2014). Preliminary evidence of salivary cortisol predicting performance in a controlled setting. Psychoneuroendocrinology.

[27] Hancock D.J., Bennett S., Roaten H., Chapman K., Stanley C. (2021). An analysis of literature on sport officiating research. Res. Q. Exerc. Sport.

[28] Anshel M.H., Kang M., Jubenville C. (2013). Sources of acute sport stress scale for sports officials: Rasch calibration. Psychol. Sport Exerc..

[29] Anshel M.H., Weinberg R.S. (1996). Coping with Acute Stress among American and Australian Basketball Referees. J. Sport Behav..

[30] World Baseball Softball Confederation (2022). 2022–2025 Official Rules of Softball—Fast Pitch. https://static.wbsc.org/assets/cms/documents/b5480455-60e2-bfc3-1e2e-3566c2765cc5.pdf.

[31] World Baseball Softball Confederation (2020). Umpire Manual Fast Pitch. https://static.wbsc.org/assets/cms/documents/cc7076b3-6edd-7331-e648-c239e70be39a.pdf.

[32] World Baseball Softball Confederation (2020). WBSC Softball Field Mechanics 3 & 4 Umpire System. https://static.wbsc.org/assets/cms/documents/71548729-ab35-3d0b-f5bd-75db2bec8216.pdf.

[33] Rainey D.W. (1995). Stress, burnout, and intention to terminate among umpires. J. Sport Behav..

[34] Guérette J., Blais C., Fiset D. (2024). Verbal aggressions against Major League Baseball umpires affect their decision making. Psychol. Sci..

[35] Coutts A.J., Reaburn P.R.J. (2000). Time and motion analysis of the AFL field umpire. J. Sci. Med. Sport.

[36] Softball Australia Levels of Accreditation. https://www.softball.org.au/levels-of-accreditation-/.

[37] Softball Australia Experience and Pass Marks Required. https://www.softball.org.au/experience-pass-marks-required/.

[38] Softball Australia Umpire Development Assessors Manual. https://cdn.revolutionise.com.au/cups/softballaust/files/edoaieetyop1mpt9.pdf.

[39] ThermoFisher Scientific Inc. Cortisol Competitive Human ELISA Kit. https://www.thermofisher.com/elisa/product/Cortisol-Competitive-Human-ELISA-Kit/EIAHCOR.

[40] Brooks Automation (2017). Freezerpro, 7.4.1-r14745.

[41] ThermoFisher Scientific Inc. Proper Sample Handling for Immunoassays Guide. https://assets.thermofisher.com/TFS-Assets/BID/Reference-Materials/proper-sample-handling-for-immunoassays-guide.pdf.

[42] Softball Australia Australian Championships Tournament Chief Umpire Manual. https://cdn.revolutionise.com.au/cups/softballaust/files/qfzdkbyjx1vybpi0.pdf.

[43] IBM Corporation (2023). IBM SPSS Statistics for Macintosh, 29.

[44] Tabachnick B.G., Fidell L.S. (2019). Using Multivariate Statistics.

[45] Pearlmutter P., DeRose G., Samson C., Linehan N., Cen Y., Begdache L., Won D., Koh A. (2020). Sweat and saliva cortisol response to stress and nutrition factors. Sci. Rep..

[46] Jones G. (1995). More than just a game: Research developments and issues in competitive anxiety in sport. Br. J. Psychol..

[47] Karageoghis C.I., Terry P.C. (2011). Inside Sport Psychology.

[48] Lai C.-P., Hsieh H.-H., Chang C.-M., Ni F.-T. (2020). The role of psychological capital in athletic performance and career development of adolescent baseball players in Taiwan. Sustainability.

[49] World Baseball Softball Confederation (2019). 2020–2021 Softball Umpire Fitness Testing Protocols. https://static.wbsc.org/assets/cms/documents/3545282d-a328-d99c-5dda-972b6d7c1445.pdf.

[50] Softball Australia Umpiring Softball Pathways. https://cdn.revolutionise.com.au/cups/softballaust/files/55ddqbznvmfijfpa.pdf.

[51] Statista GmbH Number of Softball Participants in the United States from 2010 to 2021. https://www.statista.com/statistics/191722/participants-in-softball-in-the-us-since-2006/.

